# Supplementary material for: The interactome of intact mitochondria by cross-linking mass spectrometry provides evidence for coexisting respiratory supercomplexes
Source: Mol Cell Proteomics. 2017 Dec 8;17(2):216–32. doi: 10.1074/mcp.RA117.000470 (PMC5795388; doi:10.1074/mcp.RA117.000470)
Supplement: Supplemental Data [file supp_RA117.000470_133922_0_supp_23978_fzffwf.zip › spectra_annotation/mito_spectra_annotation/179-2-29-1-3-1.pdf]

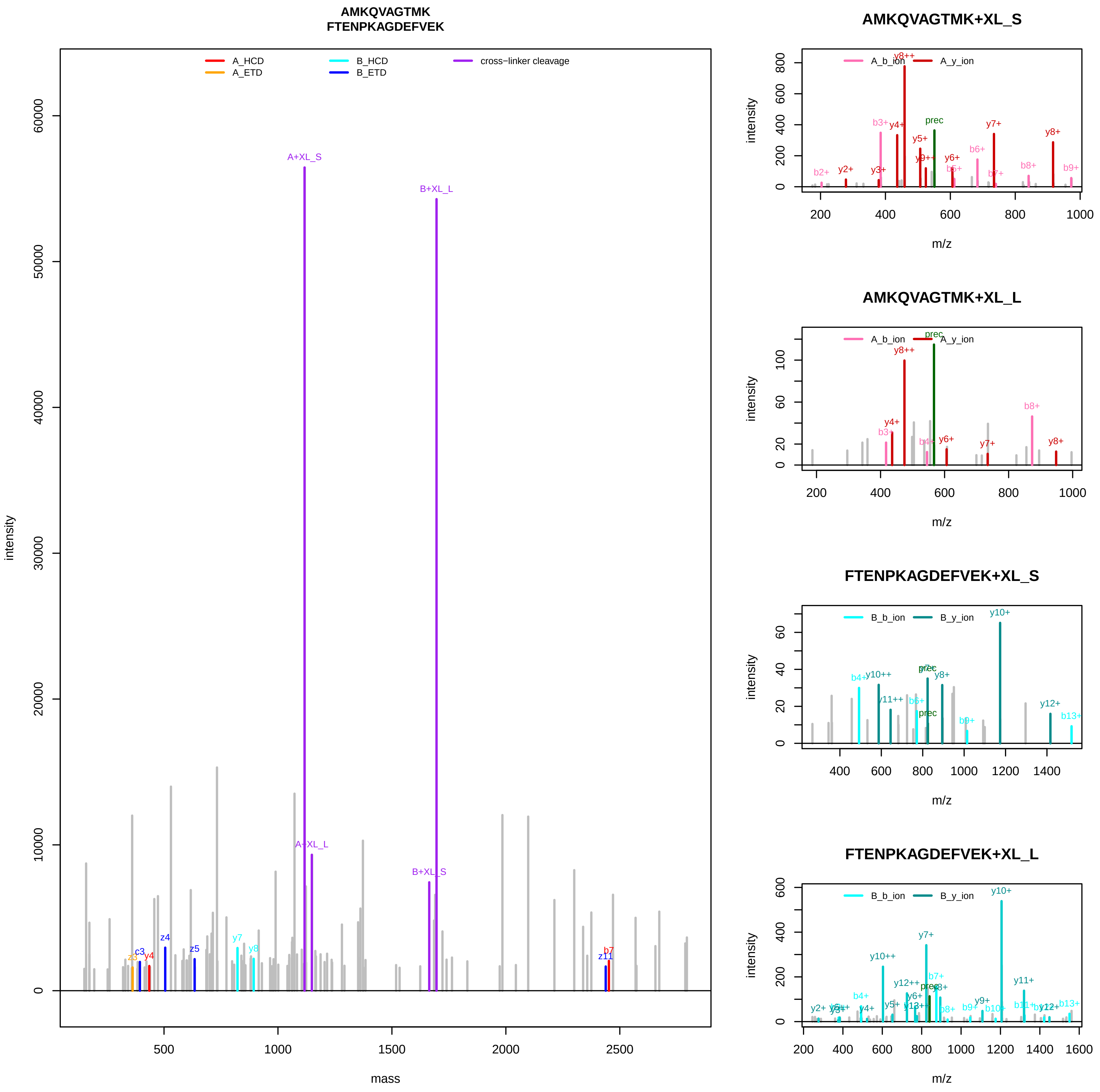
AMKQVAGTMK
FTENPKAGDEFVEK
A_HCD
A_ETD
B_HCD
B_ETD
cross−linker cleavage
A+XL_S
B+XL_L
A+XL_L
B+XL_S
z3
c3
y4
z4
z5
y7
y8
b7
z11
intensity
mass
AMKQVAGTMK+XL_S
A_b_ion
A_y_ion
y8++
b3+
y4+
prec
y7+
y8+
y5+
y9++
y6+
b6+
b2+
y2+
y3+
b5+
b7+
b8+
b9+
m/z
AMKQVAGTMK+XL_L
y8++
y4+
b3+
b4+
y6+
y7+
b8+
y8+
FTENPKAGDEFVEK+XL_S
B_b_ion
B_y_ion
y10+
b4+
y10++
y8+
y11++
b6+
b9+
y12+
b13+
FTENPKAGDEFVEK+XL_L
y10+
y7+
y10++
y12++
b7+
y8+
y11+
b4+
y4+
y5+
y6+
y9+
b9+
b10+
b11+
y12+
b13+
b8+
y2+
